# Polyurethane-Modified Epoxy Crack Sealant for Climate-Specific Asphalt Pavement Repair

**DOI:** 10.3390/polym18131617

**Published:** 2026-06-29

**Authors:** Xinmei Zhang, Biao Ma, Yan Shi, Jiafei Shu, Jianmin Liao, Tao Chen

**Affiliations:** 1 Key Laboratory for Special Area Highway Engineering of Ministry of Education, Chang’an University, Xi’an 710064, China; zhangxinmei@chd.edu.cn (X.Z.); shiyan@chd.edu.cn (Y.S.); shujiafei@chd.edu.cn (J.S.); 2 Guangxi Machinery Industry Research Institute Co., Ltd., Nanning 530007, China; liao1357liao@163.com (J.L.); chen7531chen@163.com (T.C.)

**Keywords:** polyurethane-modified epoxy, crack sealant, asphalt pavement repair, climate-specific selection, interfacial adhesion, aging resistance

## Abstract

Polyurethane-modified epoxy crack sealants can combine the cohesive strength of epoxy networks with the flexibility required for asphalt pavement crack repair. However, their selection under different winter pavement-temperature conditions requires an integrated evaluation of workability, low-temperature transition, dimensional stability, aging resistance, and interfacial adhesion. In this study, six ambient-curing polyurethane-modified epoxy crack sealants (EUPC) were prepared and assessed under representative winter pavement-temperature conditions, with SBS-modified asphalt used as a reference. All EUPC formulations satisfied the 30 min construction-window requirement, showed T_g_ values below 0 °C, T_5%_ values above 300 °C, and curing volume shrinkage no higher than 3.0%. After moisture–oxygen–ultraviolet coupled aging, the formulations retained a substantial proportion of both tensile strength and elongation, with EUPC-3/EUPC-4 showing a relatively balanced strength–ductility response. Compared with SBS-modified asphalt, the climate-matched EUPC formulations provided higher direct tensile adhesion, oblique shear adhesion, and flexural–tensile repair recovery. Overall, EUPC-1/EUPC-2, EUPC-3/EUPC-4, and EUPC-5/EUPC-6 are more suitable for mild, cold, and severe low-temperature winter conditions, respectively.

## 1. Introduction

Cracking is a common distress in asphalt pavements and can accelerate water intrusion, asphalt aging, aggregate skeleton weakening, and subsequent pavement deterioration [1,2,3,4]. Timely crack sealing is therefore widely used as a preventive maintenance strategy to extend pavement service life and improve maintenance cost-effectiveness [5,6,7]. The effectiveness of crack sealing depends on construction workability, crack-filling ability, environmental durability, and long-term adhesion between the sealant and the asphalt mixture substrate [8,9,10].

Asphalt-based, emulsion-based, and resin-based sealants have been used for pavement crack repair [8,11]. Asphalt-based sealants, including SBS-modified asphalt, are commonly used because of their compatibility with asphalt pavements and convenient construction [12,13,14,15]. Previous studies have shown that polymer modification can improve the rheological behavior, elasticity, and deformation recovery of asphalt sealants [16]. However, under winter service conditions, asphalt-based sealants may still suffer from temperature-sensitive bonding, moisture-related adhesion loss, ultraviolet aging, freeze–thaw damage, and insufficient deformation recovery [9,12,17]. Therefore, crack sealants for winter repair should be evaluated under representative pavement-temperature conditions rather than at a single standard temperature [18,19,20,21,22].

Epoxy-based repair materials have attracted attention because of their high cohesive strength, chemical resistance, and bonding potential [23,24,25,26,27]. Epoxy asphalt and epoxy-modified pavement materials have been used in surfacing layers, tack coats, bridge deck pavements, and crack-repair systems [28,29,30,31,32]. Nevertheless, conventional epoxy networks are generally stiff and brittle after curing, which may limit low-temperature deformability and crack-following ability [33,34,35]. Polyurethane modification has been reported as an effective method for improving the toughness and flexibility of epoxy systems by introducing flexible segments into the cured network [36,37,38]. For crack-sealing applications, this modification is important because the sealant must accommodate thermally induced crack movement while maintaining sufficient strength and adhesion [39,40].

Despite these advances, the evaluation of polyurethane-modified epoxy crack sealants for climate-specific pavement repair remains insufficient. First, many studies evaluate sealants at a single temperature, although adhesion and deformation behavior are strongly temperature-dependent [9,10,41]. Second, aging resistance is often assessed mainly by strength retention, whereas ductility retention is also necessary to accommodate crack movement [17,42,43,44,45]. Third, interfacial adhesion is commonly evaluated using a single loading mode, while repaired cracks experience tensile, shear, and bending-related stresses during service [23,24,44,46].

In this study, ambient-curing polyurethane-modified epoxy crack sealants, denoted as EUPC, were developed for asphalt pavement repair under representative winter pavement-temperature conditions. Six formulations were evaluated in terms of construction workability, thermal transition behavior, thermal stability, curing volume shrinkage, moisture–oxygen–ultraviolet coupled aging resistance, and interfacial adhesion. Direct tensile, oblique shear, and flexural–tensile adhesion tests were conducted using SBS-modified asphalt as the reference sealant. The objective was not to identify a universally optimal formulation, but to establish a climate-specific basis for selecting polyurethane-modified epoxy crack sealants for winter pavement repair.

## 2. Materials and Methods

### 2.1. Materials

The raw materials used to prepare EUPC are summarized in Table 1.

### 2.2. Formulation Design and Preparation of EUPC

**Figure 1:**
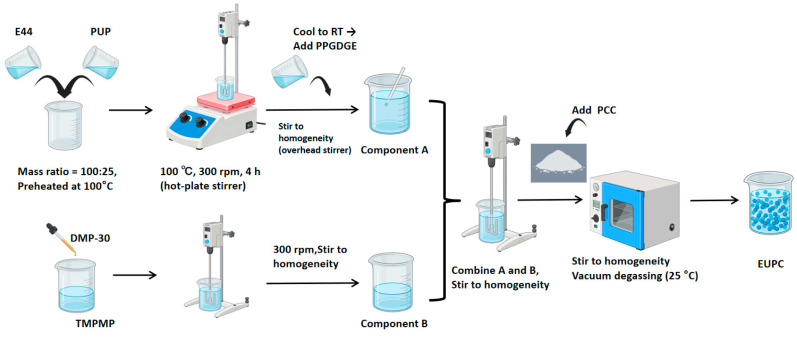
Preparation process of EUPC reactive polymer composites.

The formulations of the polyurethane-modified epoxy crack sealants, denoted as EUPC, are listed in Table 2, and the preparation procedure is shown in Figure 1. Based on the component functions in Table 1, E44 and PUP were used to construct the epoxy–urethane matrix at a fixed mass ratio of 100:25, following a previous study on epoxy resin pavement sealants [24]. The contents of PPGDGE, TMPMP, DMP-30, and PCC were adjusted to regulate workability, dimensional stability, aging resistance, and interfacial adhesion. After homogenization and vacuum degassing, all specimens were cured at ambient laboratory temperature for 7 days before testing unless otherwise specified.

### 2.3. Characterization and Testing Methods

#### 2.3.1. Construction Workability

The construction workability of the freshly prepared EUPC sealants was evaluated by monitoring the time-dependent evolution of viscosity. Rheological measurements were performed using a Discovery HR-20 hybrid rheometer (TA Instruments, New Castle, DE, USA). Immediately after mixing, each sample was loaded onto the rheometer and tested at 25 °C under a constant shear rate of 10 s^−1^. The apparent viscosity was continuously recorded as a function of time to characterize the viscosity development of the uncured sealant.

In this study, the construction window was defined as the period during which the freshly mixed sealant retained adequate flowability for handling, transportation, crack filling, and surface leveling before rapid viscosity build-up occurred. A minimum workable period of 30 min was adopted as the practical requirement for field crack-sealing operations. Therefore, the onset of rapid viscosity increase was regarded as the practical endpoint of the construction window [13,14].

#### 2.3.2. Thermal Transition and Thermal Stability

Differential scanning calorimetry (DSC) was used to determine the glass-transition temperature, T_g_, of the cured EUPC specimens. The test was performed using a DSC 200 F3 calorimeter (NETZSCH, Selb, Germany) under a nitrogen atmosphere. Approximately 5–10 mg of each cured specimen was sealed in an aluminum pan and heated from −50 to 100 °C at a heating rate of 5 °C/min. The T_g_ value was determined from the baseline shift in the DSC thermogram.

Thermogravimetric analysis (TGA) was conducted to evaluate the thermal stability of the cured EUPC specimens. The test was performed using a TG209F3 thermogravimetric analyzer (NETZSCH, Germany) under a nitrogen atmosphere. Approximately 5–10 mg of each specimen was heated from 30 to 800 °C at a heating rate of 20 °C/min. Thermal stability was assessed based on the mass-loss behavior and the 5% mass-loss temperature.

#### 2.3.3. Curing Volume Shrinkage

The curing volume shrinkage of EUPC was determined by the volume-change method to evaluate the dimensional stability and compact bonding ability of the sealant after curing. The volume of the specimen was recorded before and after curing, and the curing volume shrinkage was calculated according to Equation (1):

(1) $$S_{v}=\frac{V_{0}-V_{c}}{V_{0}}\times 100\%$$

where *S_v_* is the curing volume shrinkage (%), *V*_0_ is the initial volume before curing, and *V_c_* is the final volume after curing. Three parallel samples were tested for each group, and the average value was reported.

#### 2.3.4. Moisture–Ultraviolet Aging and Mechanical Retention

The environmental durability of cured EUPC specimens was evaluated using an accelerated moisture–ultraviolet aging test. The protocol was designed with reference to standard fluorescent UV exposure procedures for nonmetallic and polymeric materials [18,19]. Because laboratory-accelerated aging cannot be directly converted into field service life without material-specific correlation, the test was used for comparative durability assessment rather than service-life prediction [47,48].

Before aging, all specimens were cured at ambient laboratory temperature for 7 days. Aging was performed in a self-developed environmental chamber equipped with UVA-340 fluorescent lamps. The specimen surface was positioned 50 mm from the lamps, and the spectral irradiance at the specimen plane was calibrated to 0.89 W·m^−2^·nm^−1^ at 340 nm. Each aging cycle consisted of 8 h of UV irradiation at 60 ± 3 °C and 4 h of dark condensation at 50 ± 3 °C under water-saturated conditions. Aging was conducted under natural air exchange, and oxygen was not independently controlled.

Exposure durations of 7 d and 21 d were selected as two accelerated aging levels, corresponding to accumulated UV doses of 99.7 and 299.0 W·h·m^−2^·nm^−1^ at 340 nm, respectively. These levels are referred to as nominal 1-year and nominal 3-year aging levels for comparative discussion only, without implying direct equivalence to field service periods.

Tensile strength and elongation at break were measured before and after aging. Unless otherwise specified, three parallel specimens were tested for each formulation and aging condition. Mechanical property retention was calculated using Equation (2):

(2) $$R=\frac{P_{a}}{P_{0}}\times 100\%$$

where *R* is the property retention, *P*_0_ is the unaged value, and *P_a_* is the value after aging. The retention of tensile strength and elongation at break was used to evaluate the ability of the EUPC sealants to maintain strength and deformability under coupled environmental exposure [13].

#### 2.3.5. Interfacial Adhesion and Climate-Specific Applicability

Interfacial adhesion between the EUPC sealants and asphalt mixture substrates was evaluated by direct tensile, oblique shear, and flexural–tensile adhesion tests, with SBS-modified asphalt used as the reference sealant. Asphalt mixture specimens were prepared according to the required test geometries. The bonding surfaces were cleaned, dried, filled with freshly prepared sealant, and cured at ambient laboratory temperature for 7 days before testing.

The test temperatures were selected according to the temperature-based material selection concept in asphalt pavement engineering, where regional climatic conditions and representative pavement temperatures are considered for binder and repair-material evaluation [19,20,21]. Three representative winter pavement-temperature conditions were defined for climate-specific adhesion assessment, and the corresponding formulations and test temperatures are listed in Table 3. Additional oblique shear tests at 25 °C and 60 °C were conducted to evaluate interfacial shear stability under moderate and elevated pavement-temperature conditions after repair [13,41].

All adhesion tests were performed using a CMT5105 universal testing machine at a loading rate of 5 mm/min. Before testing, specimens were conditioned at the target temperature for 24 h and tested immediately under the same temperature conditions. Three parallel specimens were tested for each formulation and condition unless otherwise specified.

The strength ratio, *R*_s_, was calculated using Equation (3):

(3) $$R_{s}=\frac{F_{r}}{F_{0}}\times 100\%$$

where *R_s_* is the strength ratio, *F_r_* is the flexural–tensile adhesion strength of the repaired specimen, and *F*_0_ is the flexural strength of the original asphalt mixture specimen. Failure surfaces were visually examined to identify interfacial, cohesive, or mixed failure.

#### 2.3.6. Statistical Analysis

Unless otherwise specified, three parallel specimens were tested for each formulation and testing condition. The experimental results are reported as the mean value ± standard deviation. The coefficient of variation was calculated where appropriate to evaluate data dispersion. For comparisons among different formulations, one-way analysis of variance was performed, and differences were considered statistically significant when *p* < 0.05. For retention-related indicators, the retention values were calculated from the corresponding mean values before and after aging.

## 3. Results

### 3.1. Viscosity Evolution and Construction Workability

**Figure 2:**
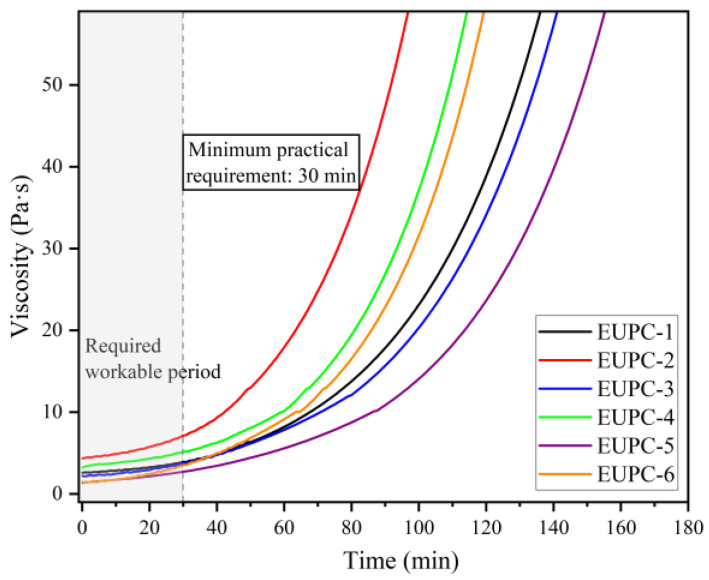
Time-dependent viscosity curves of different EUPC formulations at 25 °C.

Figure 2 shows the time-dependent apparent viscosity curves of the freshly prepared EUPC sealants at 25 °C. All formulations exhibited a typical chemorheological response of reactive polymer systems, with a low-viscosity retention stage followed by accelerated viscosity build-up. This behavior reflects the transition from an initially flowable state suitable for crack filling to a progressively structured state during curing. For epoxy-based repair and bonding systems, viscosity-related properties such as consistency, viscosity evolution, and working time are important indicators of practical applicability [13,14,16].

As shown in Figure 2, all six EUPC formulations remained in the low-viscosity retention or early build-up stage within the first 30 min after mixing. No abrupt viscosity increase occurred before the 30 min reference line. Therefore, all formulations satisfied the minimum practical construction-window requirement defined for field crack-sealing operations. This indicates that the freshly prepared EUPC sealants can provide sufficient workable time for handling, transportation, crack filling, and surface leveling under the tested conditions.

Differences in viscosity development were observed after the required workable period. EUPC-2 exhibited the earliest rapid viscosity build-up, indicating faster early structural development after mixing. In contrast, EUPC-5 maintained a lower viscosity for a longer period, suggesting better flow-retention capacity. The other formulations showed intermediate behavior. These differences are mainly associated with the combined regulation of diluent, curing agent, catalyst, and filler contents, which affects early curing kinetics and consistency development. Overall, the viscosity results confirm adequate initial workability for all EUPC formulations, while also indicating different post-placement build-up rates that may influence shape retention after crack filling.

### 3.2. Glass Transition and Thermal Decomposition Behavior

**Figure 3:**
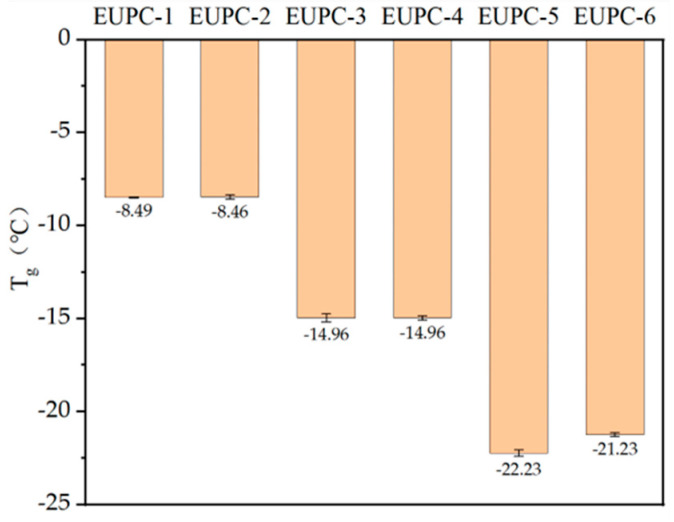
T_g_ values of cured EUPC sealants determined by DSC.

**Figure 4:**
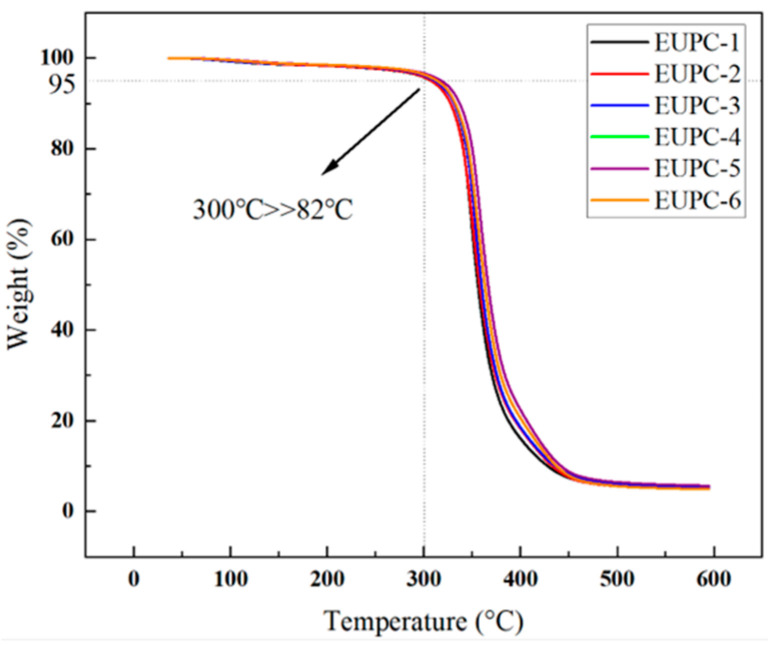
Thermogravimetric curves of different EUPC formulations. The horizontal dashed line at 95% residual mass indicates the criterion used to determine T_5%_. The EUPC-4 curve partially overlaps with other curves because of their similar mass-loss behavior.

As shown in Figure 3 and Figure 4 and Table 4, all cured EUPC sealants exhibited T_g_ values below 0 °C and T_5%_ values above 300 °C. The T_g_ values decreased from the EUPC-1/EUPC-2 group to the EUPC-5/EUPC-6 group, indicating improved low-temperature flexibility after formulation adjustment.

The T_5%_ values showed only limited differences among the formulations, confirming that all cured EUPC sealants possessed adequate thermal stability within the pavement service-temperature range. Overall, the DSC and TGA results provide thermal-performance support for the subsequent climate-specific adhesion evaluation.

### 3.3. Curing-Induced Volume Shrinkage

**Figure 5:**
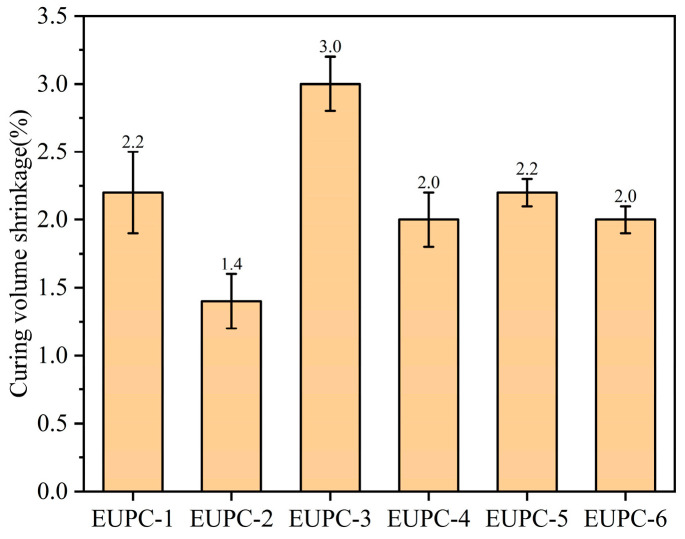
Curing volume shrinkage of cured EUPC sealants. Error bars represent standard deviations.

As shown in Figure 5 and Table 5, all cured EUPC sealants exhibited low curing volume shrinkage, with values ranging from 1.4% to 3.0%. EUPC-2 showed the lowest shrinkage, whereas EUPC-3 showed the highest value. Nevertheless, all formulations remained within a low-shrinkage range, indicating good dimensional stability after curing.

Low curing shrinkage is beneficial for crack-sealing applications because it helps maintain the filled geometry and reduce shrinkage-related defects during curing. These results provide dimensional-stability support for the subsequent adhesion and climate-specific performance evaluation.

### 3.4. Moisture–Oxygen–Ultraviolet Aging Resistance

**Figure 6:**
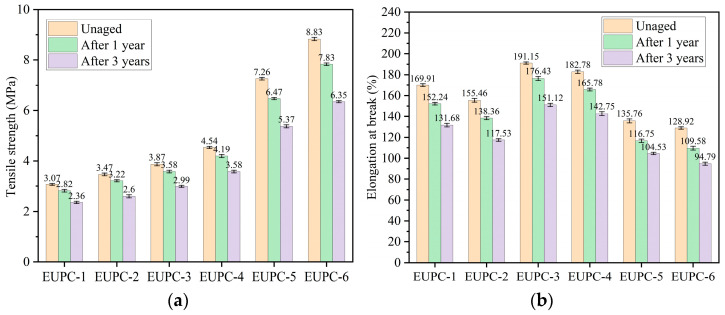
Mechanical properties of EUPC specimens with different formulations before and after coupled aging: (**a**) tensile strength and (**b**) elongation at break.

Figure 6 and Table 6 and Table 7 present the mechanical properties of the cured EUPC sealants before and after moisture–oxygen–ultraviolet coupled aging. Both tensile strength and elongation at break decreased with increasing aging duration, indicating measurable mechanical degradation under the coupled aging conditions. Nevertheless, all formulations retained a substantial proportion of their initial strength and deformation capacity after aging.

The aging response was formulation-dependent. EUPC-5 and EUPC-6 maintained higher absolute tensile strength after aging, whereas their elongation at break was comparatively lower. In contrast, EUPC-3 and EUPC-4 showed a more balanced retention of tensile strength and elongation after long-term aging. These results indicate that aging performance should be evaluated by considering both strength retention and ductility retention rather than tensile strength alone.

### 3.5. Interfacial Adhesion Under Representative Winter Conditions

**Figure 7:**
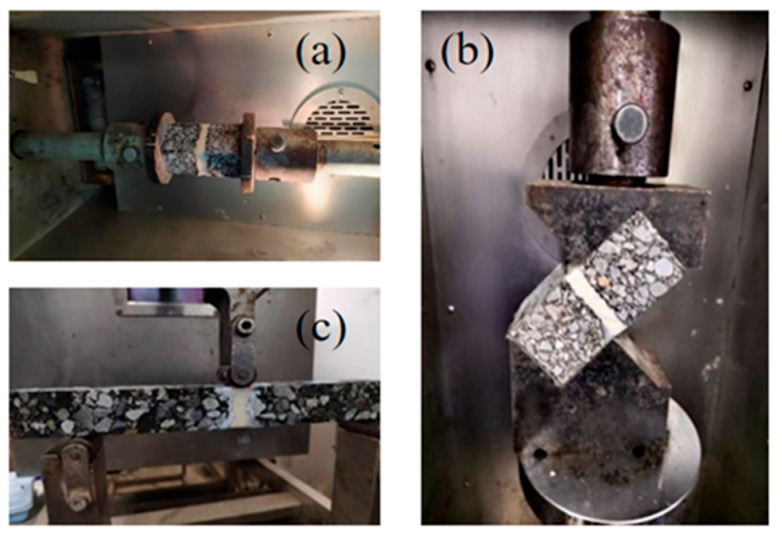
Photographs of EUPC adhesion tests: (**a**) direct tensile; (**b**) oblique shear; and (**c**) flexural–tensile.

Figure 7 shows the direct tensile, oblique shear, and flexural–tensile adhesion test configurations. The interfacial adhesion of EUPC was evaluated under representative temperatures corresponding to mild, cold, and severe low-temperature winter conditions.

#### 3.5.1. Direct Tensile Adhesion

As shown in Table 8, the direct tensile adhesion strength varied with formulation and test temperature. Under each representative winter condition, the climate-matched EUPC formulations showed higher tensile adhesion than SBS-modified asphalt. This indicates that the EUPC sealants provided stronger resistance to normal interfacial separation within their corresponding temperature ranges.

#### 3.5.2. Oblique Shear Adhesion

Table 9 presents the oblique shear adhesion strength of the repaired specimens. The EUPC formulations exhibited higher shear adhesion than SBS-modified asphalt under the corresponding winter-temperature conditions. Shear strength decreased at 25 °C and 60 °C, indicating temperature sensitivity under tangential loading; nevertheless, the cured EUPC sealants retained measurable shear resistance at elevated temperatures.

#### 3.5.3. Flexural–Tensile Adhesion

As summarized in Table 10, the EUPC-repaired specimens showed higher repaired specimen strength and strength ratio than SBS-modified asphalt under all representative winter conditions. The results indicate that the climate-matched EUPC formulations more effectively restored the load-bearing capacity of cracked asphalt mixture specimens.

## 4. Discussion

### 4.1. Formulation–Performance Relationship of EUPC Sealants

Construction workability is a key requirement for reactive crack sealants because it determines both crack-filling efficiency and early post-placement stability. Previous studies on epoxy-based repair materials have emphasized that sufficient initial flowability is necessary for substrate wetting and crack penetration, whereas subsequent viscosity build-up is required to prevent excessive flow after placement [28,34,38]. Consistent with these findings, all EUPC formulations in this study satisfied the defined construction-window requirement and then exhibited accelerated viscosity development.

The formulation-dependent viscosity evolution indicates that EUPC workability should be interpreted as a balance between workable time and shape retention, rather than simply as low initial viscosity. The differences among formulations likely resulted from the combined regulation of reactive diluent, curing agent, catalyst, and filler contents. Therefore, an appropriate EUPC formulation should provide sufficient flowability during application while developing adequate consistency after filling, which is essential for field crack-sealing operations under variable construction conditions.

### 4.2. Workability-Thermal Performance Balance

The DSC results showed that all cured EUPC formulations had T_g_ values below 0 °C, indicating favorable low-temperature transition characteristics. This agrees with previous reports that polyurethane modification and flexible molecular segments can reduce the rigidity of epoxy networks and improve low-temperature deformability [21,27,36]. In this study, the decreasing T_g_ trend from EUPC-1/EUPC-2 to EUPC-5/EUPC-6 further suggests enhanced low-temperature flexibility after formulation adjustment.

This T_g_ variation should be regarded as the integrated effect of the formulation design because the reactive diluent, curing system, catalyst, and PCC content were adjusted simultaneously. The TGA results showed that all formulations had T_5%_ values above 300 °C, confirming adequate thermal stability within the pavement service-temperature range. However, T_g_ and T_5%_ should be considered supporting indicators rather than independent criteria for climate-specific selection. Final formulation applicability should be evaluated together with workability, dimensional stability, aging resistance, and interfacial adhesion [25,26,36,39].

### 4.3. Curing Volume Shrinkage and Interfacial Reliability

Curing shrinkage is an important factor affecting the service reliability of polymer-based crack sealants. Previous studies on epoxy repair materials have indicated that curing contraction can generate interfacial stress when the material is confined by rigid substrates. In pavement crack repair, this effect may increase the risk of interfacial debonding, edge separation, or void formation along the crack wall.

The low shrinkage values obtained in this study indicate that the EUPC sealants maintained favorable dimensional stability after curing. This behavior was likely associated with the combined formulation design, including the epoxy–urethane network and PCC incorporation. The filler phase may reduce the volume fraction of the reactive resin matrix and restrict curing contraction, while the polymer network maintains continuity after curing. Because several formulation variables were adjusted simultaneously, the shrinkage behavior should be interpreted as the integrated effect of the composite system rather than the contribution of a single component [49,50].

These dimensional-stability results help explain the subsequent adhesion performance. Lower curing shrinkage can reduce shrinkage-induced defects and help maintain contact between the sealant and the crack wall before full interfacial strength develops. Therefore, curing shrinkage should be considered together with workability, thermal transition behavior, aging resistance, and interfacial adhesion when evaluating the climate-specific applicability of EUPC sealants.

### 4.4. Aging Resistance and Strength–Ductility Balance

Environmental aging of polymer-based crack sealants is generally associated with moisture diffusion, oxidation, ultraviolet-induced degradation, and possible post-curing, which can reduce strength and deformability [17,43]. The decreases in tensile strength and elongation at break observed in this study are consistent with these reported aging effects. Different from studies focusing mainly on residual strength, the present evaluation emphasizes the coupled retention of strength and ductility, which is more relevant to crack-sealing materials subjected to thermal crack movement [42,44,45].

The strength–ductility balance is important because high residual strength alone does not ensure repair reliability. A sealant with insufficient deformation capacity may be more susceptible to brittle damage or interfacial stress concentration, whereas excessive flexibility without adequate strength may weaken load transfer and bonding stability. In this study, EUPC-5 and EUPC-6 retained higher tensile strength after aging, while EUPC-3 and EUPC-4 showed a more balanced mechanical response, indicating better compatibility between load-bearing capacity and deformation tolerance.

The observed aging behavior likely reflects the integrated response of the epoxy–urethane network, reactive diluent, curing system, catalyst dosage, and PCC incorporation. Therefore, the aging resistance cannot be assigned to a single component. For climate-specific pavement crack repair, suitable EUPC formulations should retain sufficient strength while maintaining adequate ductility after environmental aging, providing a durability basis for the subsequent adhesion evaluation under representative winter pavement-temperature conditions.

### 4.5. Temperature-Dependent Interfacial Adhesion and Climate-Specific Selection

Interfacial adhesion is a key performance criterion for crack sealants because repair failure often initiates at the sealant–crack wall interface under thermal contraction, crack opening, and traffic-induced stress. Previous studies have shown that the adhesion of asphalt-based and polymer-based sealants is affected by temperature, substrate wetting, sealant stiffness, and mechanical compatibility with asphalt mixtures [23,24,51]. The present results are consistent with this understanding, as the direct tensile, oblique shear, and flexural–tensile adhesion responses varied with both formulation and test temperature.

Compared with SBS-modified asphalt, the EUPC formulations showed stronger adhesion within their corresponding winter pavement-temperature ranges. This improvement was likely associated with the combined effects of reactive curing, low curing shrinkage, and formulation-dependent flexibility. However, the reduction in shear strength at 25 °C and 60 °C indicates that the repaired interface remained temperature-sensitive, especially under tangential loading.

The three adhesion tests provide complementary information for formulation selection. Direct tensile testing reflects resistance to normal interfacial separation, oblique shear testing reflects resistance to combined normal and tangential stress, and flexural–tensile testing evaluates the load-bearing recovery of repaired asphalt mixture specimens. Therefore, climate-specific applicability should be determined from the combined adhesion response rather than from a single strength value. Based on this principle, EUPC-1/EUPC-2 are more suitable for mild winter conditions, EUPC-3/EUPC-4 for cold winter conditions, and EUPC-5/EUPC-6 for severe low-temperature winter conditions.

### 4.6. Broader Implications, Limitations, and Future Research

The results suggest that reactive EUPC sealants can be selected according to representative winter pavement-temperature conditions rather than treated as a universal formulation. This application-oriented approach is consistent with temperature-based material selection in asphalt pavement engineering and highlights the need to evaluate workability, thermal transition behavior, curing shrinkage, aging resistance, and interfacial adhesion together.

Several limitations should be noted. The representative winter conditions were defined using selected laboratory temperatures and may not fully capture field temperature histories. The coupled aging protocol did not include freeze–thaw cycles, deicing salts, traffic loading, or repeated crack movement. In addition, because multiple formulation variables were adjusted simultaneously, the individual contribution of each component could not be quantified independently.

Future research should include long-term field validation, cyclic thermal–mechanical loading, freeze–thaw and salt exposure, and interface-level failure characterization. Factorial or response-surface experimental designs would also help clarify the individual and interactive effects of polyurethane modification, reactive diluent, curing system, catalyst dosage, and PCC content on EUPC performance.

## 5. Conclusions

In this study, an ambient-curing epoxy–urethane/precipitated calcium carbonate composite crack sealant, denoted as EUPC, was developed for asphalt pavement crack repair. The main conclusions are as follows:

1. All EUPC formulations met the defined construction-window requirement and showed controllable viscosity development, indicating adequate workability for crack filling and post-placement stability.
2. The cured EUPC sealants exhibited T_g_ values below 0 °C, T_5%_ values above 300 °C, and low curing volume shrinkage, confirming favorable low-temperature transition behavior, thermal stability, and dimensional stability.
3. After moisture–oxygen–ultraviolet coupled aging, the EUPC sealants retained both tensile strength and elongation at break, indicating that aging resistance should be assessed using a strength–ductility balance rather than strength retention alone.
4. Compared with SBS-modified asphalt, the climate-matched EUPC formulations showed higher tensile adhesion, shear adhesion, and flexural–tensile repair recovery. EUPC-1/EUPC-2 are more suitable for mild winter conditions, EUPC-3/EUPC-4 for cold winter conditions, and EUPC-5/EUPC-6 for severe low-temperature winter conditions.

## Figures and Tables

**Table 1:** Properties of raw materials.

Function	Raw Material	Properties	Results
Resin matrix	E44 (Nantong Xingchen Synthetic Material Co., Ltd., Nantong, China)	Viscosity (mPa·s, 25 °C)	18,000–25,000
		Density (g/mL, 25 °C)	1.17
		Epoxy Value (eq/100 g)	0.42–0.46
Toughener	PUP (Shandong Yiborun New Materials Technology Co., Ltd., Binzhou, China)	Viscosity (mPa·s, 80 °C)	250–300
		Density (g/mL, 25 °C)	1.16
Curing agent	TMPMP (Guangzhou Geling New Materials Co., Ltd., Guangzhou, China)	Viscosity (mPa·s, 25 °C)	135–165
		Density (g/mL, 25 °C)	1.15–1.30
Diluent	PPGDGE (Guangzhou Yinghong Chemical Co., Ltd., Guangzhou, China)	Viscosity (mPa·s, 25 °C)	40–70
		Density (g/mL, 25 °C)	1.05
		Epoxy Value (eq/100 g)	0.28–0.36
Catalyst	DMP-30 (Guangzhou Geling New Materials Co., Ltd., Guangzhou, China)	Viscosity (mPa·s, 25 °C)	80–150
		Density (g/mL, 25 °C)	0.98
		Amine Value, mgKOH/g	600 ± 20
Filler	PCC (Hebei Hongyao Mineral Products Processing Co., Ltd., Shijiazhuang, China)	Particle Size (D_50_, μm)	2.5–3.5
		Bulk Density (g/cm^3^, 25 °C)	1.2
		CaCO_3_ Purity (%)	≥98.5

**Table 2:** Detailed formulation of EUPC reactive composites per cubic meter.

Specimen	E44	PUP	PPGDGE	TMPMP	DMP-30	PCC
EUPC-1	100	25	80	74.1	2.8	88.0
EUPC-2	100	25	80	76.7	2.8	112.0
EUPC-3	100	25	100	80.2	3.0	92.4
EUPC-4	100	25	100	83.7	3.0	123.1
EUPC-5	100	25	140	86.5	3.6	108.1
EUPC-6	100	25	140	94.0	3.6	145.0

Note: The values are expressed as parts by mass, with E44 fixed at 100 parts. The mass ratio of E44 to PUP was fixed at 100:25 for all formulations [24].

**Table 3:** Representative pavement-temperature conditions used for climate-specific adhesion evaluation.

Representative Condition	Formulation	Direct Tensile Test Temperatures	Oblique Shear Test Temperatures
Mild winter condition	EUPC-1	−5, 0, 15 °C	−5, 0, 15, 25, 60 °C
	EUPC-2		
Cold winter condition	EUPC-3	−15, −10, −5 °C	−15, −10, −5, 25, 60 °C
	EUPC-4		
Severe low-temperature winter condition	EUPC-5	−25, −20, −15 °C	−25, −20, −15, 25, 60 °C
	EUPC-6		

**Table 4:** Thermal transition and thermal-stability parameters of cured EUPC sealants.

Specimen	EUPC-1	EUPC-2	EUPC-3	EUPC-4	EUPC-5	EUPC-6
T_g_/°C	−8.49 ± 0.03	−8.46 ± 0.12	−14.96 ± 0.21	−14.96 ± 0.11	−22.23 ± 0.18	−21.23 ± 0.09
T_5%_/°C	305.1 ± 0.21	306.1 ± 0.13	311.2 ± 0.03	313.1 ± 0.22	307.6 ± 0.03	307.9 ± 0.04

**Table 5:** Curing volume shrinkage results of different EUPC formulations.

Formulation	EUPC-1	EUPC-2	EUPC-3	EUPC-4	EUPC-5	EUPC-6
Initial volume (mL)	5	5	5	5	5	5
Volume change after curing (mL)	0.11 ± 0.02	0.07 ± 0.01	0.15 ± 0.02	0.10 ± 0.01	0.11 ± 0.01	0.10 ± 0.01
curing volume shrinkage (%)	2.2 ± 0.3	1.4 ± 0.2	3.0 ± 0.2	2.0 ± 0.2	2.2 ± 0.1	2.0 ± 0.1

Note: Values are presented as mean ± standard deviation. The initial volume of each uncured specimen was 5 mL.

**Table 6:** Tensile strength and strength retention of cured EUPC sealants before and after moisture–oxygen–ultraviolet coupled aging.

Aging Condition	EUPC-1	EUPC-2	EUPC-3	EUPC-4	EUPC-5	EUPC-6
Unaged tensile strength (MPa)	3.07 ± 0.04	3.47 ± 0.05	3.87 ± 0.06	4.54 ± 0.04	7.26 ± 0.05	8.83 ± 0.06
Tensile strength after 1 year (MPa)	2.82 ± 0.05	3.22 ± 0.04	3.58 ± 0.05	4.19 ± 0.06	6.47 ± 0.04	7.83 ± 0.05
Tensile strength after 3 years (MPa)	2.36 ± 0.04	2.60 ± 0.06	2.99 ± 0.04	3.58 ± 0.05	5.37 ± 0.06	6.35 ± 0.04
Retention after 1 year (%)	91.86 ± 0.65	92.80 ± 0.72	92.51 ± 0.68	92.29 ± 0.70	89.12 ± 0.66	88.67 ± 0.73
Retention after 3 years (%)	76.87 ± 0.71	74.93 ± 0.66	77.26 ± 0.73	78.85 ± 0.67	73.97 ± 0.72	71.91 ± 0.68

**Table 7:** Elongation at break and elongation retention of different EUPC formulations before and after coupled aging.

Aging Condition	EUPC-1	EUPC-2	EUPC-3	EUPC-4	EUPC-5	EUPC-6
Unaged elongation at break (%)	169.91 ± 1.32	155.46 ± 1.75	191.15 ± 1.08	182.78 ± 1.54	135.76 ± 1.88	128.92 ± 1.21
Elongation after 1 year (%)	152.24 ± 1.15	138.36 ± 1.41	176.43 ± 1.86	165.78 ± 1.29	116.75 ± 1.63	109.58 ± 1.77
Elongation after 3 years (%)	131.68 ± 1.67	117.53 ± 1.23	151.12 ± 1.52	142.75 ± 1.91	104.53 ± 1.11	94.79 ± 1.45
Retention after 1 year (%)	89.60 ± 0.54	89.00 ± 0.68	92.30 ± 0.42	90.70 ± 0.75	86.00 ± 0.61	85.00 ± 0.83
Retention after 3 years (%)	77.50 ± 0.72	75.60 ± 0.46	79.06 ± 0.81	78.10 ± 0.59	77.00 ± 0.87	73.53 ± 0.49

Note: Values are presented as mean ± standard deviation.

**Table 8:** Direct tensile adhesion strength of EUPC sealants and SBS-modified asphalt under representative winter conditions.

Climatic Zone	Temperature (°C)	EUPC-1	EUPC-2	EUPC-3	EUPC-4	EUPC-5	EUPC-6	SBS-Modified Asphalt
Mild winter condition	−5	2.24 ± 0.07	2.38 ± 0.03	—	—	—	—	1.43 ± 0.02
	0	1.48 ± 0.03	1.63 ± 0.11	—	—	—	—	1.28 ± 0.05
	15	1.21 ± 0.06	1.27 ± 0.03	—	—	—	—	0.89 ± 0.07
Cold winter condition	−15	—	—	2.97 ± 0.01	3.12 ± 0.03	—	—	1.49 ± 0.09
	−10	—	—	2.86 ± 0.07	2.94 ± 0.02	—	—	2.28 ± 0.04
	−5	—	—	2.34 ± 0.06	2.45 ± 0.04	—	—	1.43 ± 0.02
Severe low-temperature winter condition	−25	—	—	—	—	2.76 ± 0.08	2.83 ± 0.07	0.52 ± 0.03
	−20	—	—	—	—	3.37 ± 0.13	3.44 ± 0.10	0.87 ± 0.02
	−15	—	—	—	—	2.84 ± 0.09	3.01 ± 0.11	1.49 ± 0.09

Note: Values are expressed as mean ± SD. “—” indicates that the formulation was not tested under the corresponding condition.

**Table 9:** Oblique shear adhesion strength of EUPC sealants and SBS-modified asphalt under representative winter conditions.

Climatic Zone	Temperature (°C)	EUPC-1	EUPC-2	EUPC-3	EUPC-4	EUPC-5	EUPC-6	SBS-Modified Asphalt
Mild winter condition	−5	6.74 ± 0.17	7.12 ± 0.12	—	—	—	—	1.26 ± 0.07
	0	4.22 ± 0.07	4.89 ± 0.09	—	—	—	—	1.02 ± 0.04
	15	2.74 ± 0.02	3.56 ± 0.11	—	—	—	—	0.76 ± 0.06
	25	1.26 ± 0.03	2.08 ± 0.12	—	—	—	—	0.32 ± 0.02
	60	0.78 ± 0.03	1.06 ± 0.08	—	—	—	—	—
Cold winter condition	−15	—	—	8.12 ± 0.11	8.86 ± 0.13	—	—	5.64 ± 0.11
	−10	—	—	6.89 ± 0.09	7.34 ± 0.14	—	—	3.58 ± 0.15
	−5	—	—	6.02 ± 0.07	6.74 ± 0.19	—	—	1.26 ± 0.07
	25	—	—	1.33 ± 0.05	1.56 ± 0.11	—	—	0.32 ± 0.02
	60	—	—	0.72 ± 0.07	0.84 ± 0.04	—	—	—
Severe low-temperature winter condition	−25	—	—	—	—	6.78 ± 0.15	6.54 ± 0.14	0.89 ± 0.03
	−20	—	—	—	—	8.98 ± 0.21	9.87 ± 0.16	1.38 ± 0.08
	−15	—	—	—	—	7.32 ± 0.24	8.14 ± 0.07	5.64 ± 0.11
	25	—	—	—	—	1.45 ± 0.12	1.72 ± 0.13	0.32 ± 0.03
	60	—	—	—	—	0.76 ± 0.01	0.85 ± 0.07	—

Note: Values are expressed as mean ± SD. “—” indicates that the formulation was not tested under the corresponding condition. Tests at 25 °C and 60 °C were conducted to evaluate shear stability under moderate and elevated pavement-temperature conditions.

**Table 10:** Flexural–tensile adhesion performance of repaired asphalt mixture specimens under representative winter conditions.

**(a) Mild winter condition**			
**Parameter**	**EUPC-1**	**EUPC-2**	**SBS-Modified Asphalt**
Original specimen strength (MPa)	8.03 ± 0.25	8.24 ± 0.31	8.13 ± 0.55
Repaired specimen strength (MPa)	5.28 ± 0.23	5.34 ± 0.15	2.18 ± 0.26
Strength ratio (%)	65.8 ± 3.5	64.8 ± 3.0	26.8 ± 3.7
**(b) Cold winter condition**			
**Parameter**	**EUPC-3**	**EUPC-4**	**SBS-Modified Asphalt**
Original specimen strength (MPa)	10.15 ± 0.53	10.23 ± 0.71	10.08 ± 0.44
Repaired specimen strength (MPa)	8.26 ± 0.47	8.70 ± 0.45	4.32 ± 0.26
Strength ratio (%)	81.4 ± 6.3	85.8 ± 7.4	42.9 ± 3.2
**(c) Severe low-temperature winter condition**			
**Parameter**	**EUPC-5**	**EUPC-6**	**SBS-Modified Asphalt**
Original specimen strength (MPa)	15.34 ± 0.83	15.21 ± 0.51	14.98 ± 0.42
Repaired specimen strength (MPa)	9.78 ± 0.33	10.65 ± 0.21	0.94 ± 0.15
Strength ratio (%)	63.8 ± 4.1	70.0 ± 2.7	6.30 ± 1.0

Note: Values are expressed as mean ± SD. The strength ratio was calculated as the ratio of repaired specimen strength to original specimen strength. The SD of the strength ratio was estimated by error propagation.

## Data Availability

The original contributions presented in this study are included in the article. Further inquiries can be directed to the corresponding author.

## Abbreviations

The following abbreviations are used in this manuscript:

EUPC	Ambient-curing epoxy–urethane/precipitated calcium carbonate reactive polymer composite
E44	E44 epoxy resin
PUP	Isocyanate-terminated polyether polyurethane prepolymer
TMPMP	Trimethylolpropane tris(3-mercaptopropionate)
PPGDGE	Poly(propylene glycol) diglycidyl ether
DMP-30	2,4,6-Tris(dimethylaminomethyl)phenol
PCC	Precipitated calcium carbonate
SBS	Styrene–butadiene–styrene
DSC	Differential scanning calorimetry
TGA	Thermogravimetric analysis
T_g_	Glass-transition temperature
T_5%_	Temperature at 5% mass loss
D_50_	Median particle size
